# Endovascular dual internal-external iliac balloon occlusion creates a reproducible porcine survival model of unilateral acute hindlimb ischemia

**DOI:** 10.1016/j.jvssci.2026.100432

**Published:** 2026-07-14

**Authors:** Ariyan Tabesh, Sahil Patel, Tripti Mathur, Priya Dhawan, Zosia Zawacki, Diana Bauer, Pedro Ruivo, Rachel M. Russo, Shahram Aarabi

**Affiliations:** a Department of Surgery, University of California San Francisco, San Francisco, CA; b Department of Neurology, University of California San Francisco, San Francisco, CA; c Laboratory Animal Resource Center, University of California San Francisco, San Francisco, CA; d Department of Veterinary Medicine, University of California Davis, Davis, CA; e Department of Surgery, University of California Davis, Davis, CA

**Keywords:** Acute limb ischemia, Porcine model, Endovascular balloon occlusion, Ischemia-reperfusion injury, Translational research, Large animal model

## Abstract

**Objective:**

Large-animal models are essential for studying acute limb ischemia (ALI), yet most existing porcine ALI models are nonsurvival designs intended for short-term physiologic or metabolic investigation. In contrast, current survival models predominantly replicate chronic limb ischemia by surgically ligating the external iliac artery (EIA) or common femoral artery (CFA), an approach that does not generate a complete acute ischemic insult. In our previous studies, single-segment occlusion of the external iliac artery or CFA failed to produce profound unilateral ischemia because of robust collateral reconstitution through the internal iliac system and pelvic vasculature. To address this limitation, we developed a reproducible, minimally invasive survival model of unilateral hindlimb ALI using simultaneous balloon occlusion of the ipsilateral internal artery and EIA (ie, “dual internal-external iliac balloon occlusion”), enabling reliable 7-day survival and longitudinal assessment of functional, electrophysiologic, and histologic outcomes of ALI.

**Methods:**

Female Yorkshire pigs (50-55 kg) underwent general anesthesia, surgical exposure and vascular access of the common carotid artery, and percutaneous vascular access of the CFA using the Seldinger technique. Angiography confirmed the characteristic porcine anatomy, demonstrating an aortic trifurcation with right and left EIAs and a single internal iliac trunk providing extensive collateral flow. Iterative testing compared tourniquet application, distal aortic balloon occlusion, selective external iliac balloon occlusion, and simultaneous EIA and internal iliac artery balloon occlusion. Sustained 6-hour unilateral hindlimb ischemia was ultimately achieved with balloon occlusion of both the external and internal iliac arteries and validated by angiography, Doppler ultrasound study, and pulse oximetry. Primary outcomes included Tarlov gait score, electrophysiologic testing, and blinded terminal histologic analysis of peroneus tertius muscle and tibial nerve at 7 days postoperatively.

**Results:**

Dual internal-external iliac balloon occlusion consistently produced complete hindlimb ischemia for 6 hours without mortality or systemic morbidity. Pulse oximetry and Doppler ultrasound study confirmed absent distal flow during occlusion, with angiographic validation of no reconstitution. Pigs survived to day 7, exhibiting immediate postoperative functional impairment. Histology showed acute ischemic injury with myofiber necrosis, inflammatory infiltration, coagulative necrosis, mineralization and architectural disruption. Nerve conduction studies demonstrated complete loss of compound muscle action potentials in most specimens.

**Conclusions:**

In our porcine survival model, a minimally invasive dual internal-external iliac balloon occlusion technique consistently induced profound hindlimb ischemia consistent with prolonged ALI. The resulting ischemic insult was validated through longitudinal assessment of gait function, neuroelectrophysiologic testing, and terminal histopathologic analysis. This model provides a robust representation of human ALI, enabling study of its long-term functional and histological deficits. By avoiding laparotomy and open arterial clamping, our model minimizes additional surgical insult, reduces systemic injury and eliminates confounding collateral flow.

**Clinical Relevance:**

Acute limb ischemia is a vascular emergency that can lead to limb loss, long-term disability, and death if not treated promptly. Progress in this field has been limited in part by the lack of a reliable large-animal survival model that reproduces severe acute ischemia. In swine, single-vessel occlusion often fails because pelvic collateral vessels restore distal blood flow. In this study, we developed a minimally invasive dual internal-external iliac balloon occlusion model that produces consistent unilateral hindlimb ischemia with measurable functional, electrophysiologic, and histologic injury. This model may be useful for studying ischemia-reperfusion injury and testing new therapies for acute limb ischemia.

**Article Highlights**

• **Type of Research:** Single-center prospective experimental longitudinal survival study
• **Key Findings:** In 15 female Yorkshire swine undergoing 6-hour unilateral hindlimb ischemia via dual internal-external iliac balloon occlusion, complete distal perfusion loss was sustained without mortality. Mean gait scores declined from 4.0 to ∼2.5 by postoperative day 7. Histologic muscle injury scores were markedly higher in ischemic vs control limbs (17.7 vs 3.3).
• **Take Home Message:** Simultaneous endovascular occlusion of the internal and external iliac arteries produces a reproducible, minimally invasive survival model of porcine unilateral acute limb ischemia with durable functional and tissue-level deficits, providing a translational large-animal platform for studying ischemia-reperfusion injury.

Acute limb ischemia (ALI) is a time-critical vascular emergency marked by an abrupt reduction in limb perfusion that threatens viability and demands urgent revascularization. Incidence is ∼6 to 12 per 100,000 annually and 1-year mortality approaches 40%, underscoring the need for translational models that capture human-relevant pathophysiology and allow controlled ischemia-reperfusion studies.^1^, ^2^, ^3^, ^4^, ^5^

Large-animal models are indispensable for investigating ALI and ischemia-reperfusion injury because they recapitulate human-like vascular anatomy, physiology, and recovery patterns that rodent systems cannot fully reproduce. Swine share key similarities with humans in limb vascular architecture, muscle composition and peripheral nerve structure, making them uniquely suited for studying the tissue-level consequences of ALI. Porcine skeletal muscle tolerates relatively prolonged warm ischemia in experimental models, often remaining partially viable after 4 to 8 hours, with reported critical ischemia times approaching 9 to 10 hours^6^^,^^7^ Classical human data suggest irreversible muscle injury begins after approximately 2 to 3 hours^8^ A 6-hour ischemic interval in pigs should therefore not be interpreted as temporally equivalent to 6 hours in humans, but rather as producing a degree of myonecrosis that lies within the clinical spectrum of ALI and may approximate the injury seen after a shorter ischemic duration in patients.

Given this physiology, we sought to develop a survival model of complete acute unilateral hindlimb ischemia. Existing porcine models of ALI proved inadequate for this purpose because many are nonsurvival designs intended only for short-term physiologic or metabolic endpoints, precluding longitudinal assessment. Conversely, current survival models primarily mimic chronic limb-threatening ischemia, relying on surgical ligation of the external or CFA, an approach that fails to generate sustained, profound ischemia because robust collateral flow from the internal iliac system rapidly reconstitutes distal perfusion, a finding we confirmed angiographically. To overcome these limitations, we developed a novel, minimally invasive survival model using simultaneous balloon occlusion of both the internal and external iliac arteries (EIAs), producing reproducible, complete ischemia with controlled reperfusion and enabling longitudinal functional, electrophysiologic, and histopathologic evaluation.

## Methods

### Animal care and model overview

All procedures were performed under protocols approved by the Institutional Animal Care and Use Committee (IACUC) at the University of California, San Francisco. Animals were housed and cared for in American Association for the Accreditation of Laboratory Animal Care-accredited facilities at the University of California, San Francisco Laboratory Animal Resource Center in accordance with the Guide for the Care and Use of Laboratory Animals. This study is reported in accordance with the Animal Research: Reporting of In Vivo Experiments (ARRIVE 2.0) guidelines for reporting animal research.

Fifteen female Yorkshire swine (n = 15; 50-55 kg) were used to develop and validate the survival model of unilateral hindlimb ischemia. Animals had free access to water and standard chow. Female Yorkshire swine were used because female animals are commonly preferred in large-animal survival models due to their less aggressive behavior, ease of handling, and lower risk of postoperative injury to other animals. Female animals were also the only sex approved under the University of California, San Francisco Laboratory Animal Resource Center protocol for this study. The 15 animals were allocated across the iterative model-development process as follows: tourniquet application was tested in two animals, distal aortic balloon occlusion in one animal, isolated EIA balloon occlusion in one animal, and dual internal-external iliac balloon occlusion in 11 animals. The animal that underwent distal aortic balloon occlusion was euthanized early on postoperative day (POD) 1 because of inability to ambulate and meeting prespecified humane endpoint criteria.

### Anesthesia and perioperative management

Animals were premedicated with intramuscular ketamine (20-25 mg/kg) and midazolam (0.5 mg/kg). Following intravenous (IV) access, anesthesia was induced with propofol (1-2 mg/kg IV) and maintained with inhaled isoflurane (1%-3%). Endotracheal intubation was performed, and animals were placed on volume-controlled ventilation. Continuous physiologic monitoring included electrocardiogram, pulse oximetry, capnography, and invasive arterial blood pressure.

### Endovascular access and angiography

**Fig 1:**
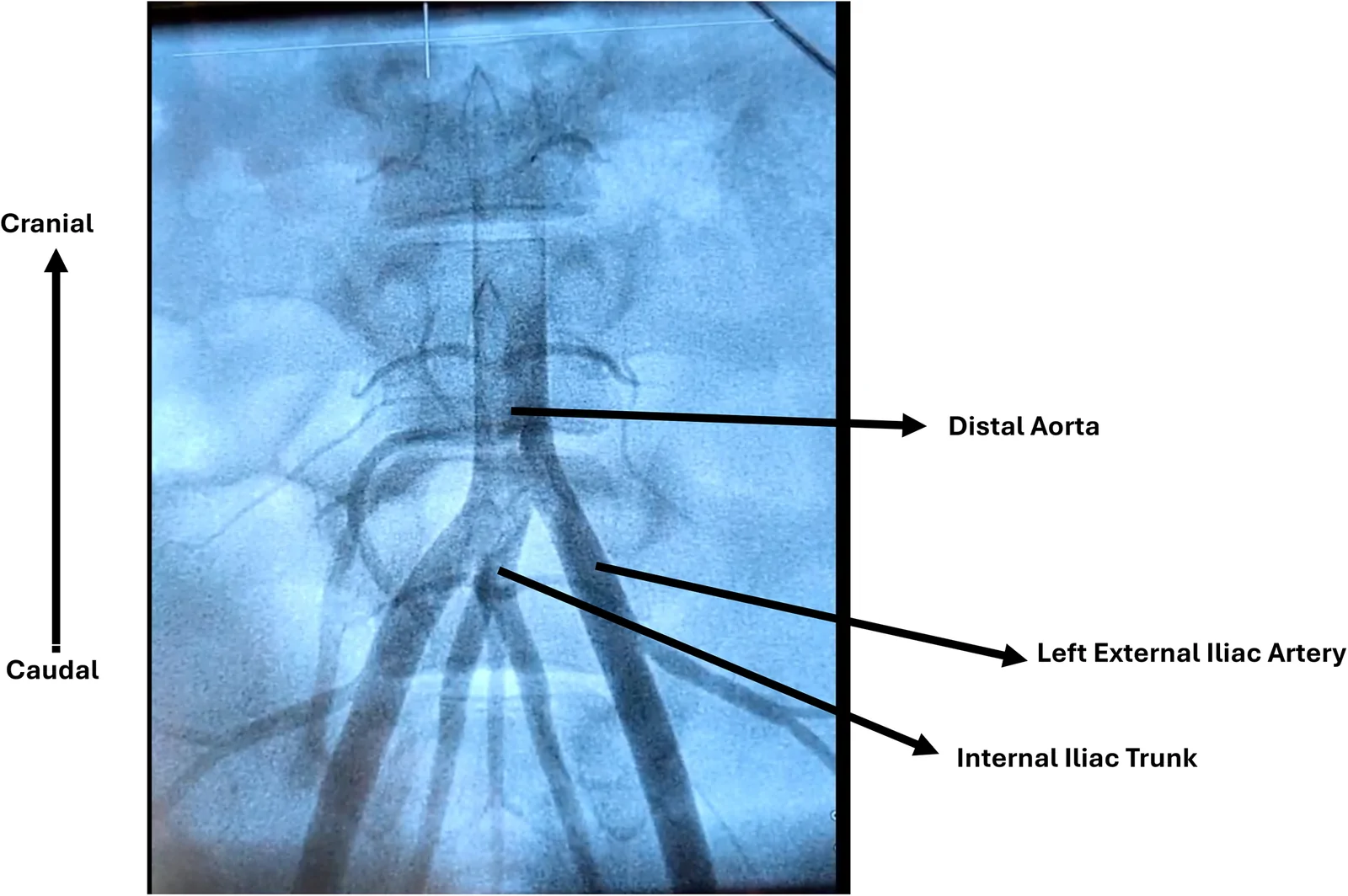
Angiography showing baseline aortoiliac anatomy in swine. The distal aorta terminates in a trifurcation that gives rise to the right and left external iliac arteries and a single internal iliac trunk, which subsequently branches into the right and left internal iliac arteries.

The left carotid artery was surgically dissected and cannulated with a 12F vascular sheath (Cook Medical), and the left internal jugular vein was dissected and cannulated with a 7F catheter for fluid and medication administration, resuscitation, and blood collection (Terumo). The left femoral artery was accessed percutaneously under ultrasound guidance and cannulated with a 7F catheter (Terumo). Three access sites were required because each served a distinct function during the dual internal-external iliac balloon occlusion protocol. The left internal jugular vein served as central venous access for laboratory sampling, medication administration, and fluid resuscitation. The left femoral arterial access site was used to deploy the balloon catheter into the left EIA. The left carotid arterial access site was used to deploy the balloon catheter into the left internal iliac artery (IIA). This access strategy allowed simultaneous occlusion of the ipsilateral EIA and IIA while preserving central venous access throughout the ischemia-reperfusion protocol. Systemic heparinization was initiated with 100 U/kg IV. Fluoroscopic angiography was performed using iodinated contrast. Baseline angiograms confirmed the characteristic porcine anatomy: absence of common iliac arteries, with the distal aorta trifurcating directly into both EIAs and a single, dominant internal iliac trunk (Fig 1).

### Iterative development of an acute limb ischemia model

We evaluated four occlusion strategies sequentially to determine their ability to generate complete, sustained unilateral hindlimb ischemia. The advantages and limitations of each iterative approach are outlined in Table I and summarized below. Strategies were not repeated when they failed to meet the core design requirements of the model based on direct physiologic, functional, or angiographic evidence. Distal aortic balloon occlusion was not repeated because it produced bilateral hindlimb ischemia, precluded use of the contralateral limb as an internal control, and resulted in severe postoperative ambulatory impairment requiring early euthanasia. Isolated EIA occlusion was not repeated because serial angiography demonstrated rapid collateral reconstitution of distal limb perfusion through the internal iliac and pelvic collateral network despite persistent balloon occlusion of the EIA. Thus, this strategy was considered anatomically unable to produce the intended endpoint of sustained complete unilateral ischemia. Bilateral IIA occlusion was not performed because ipsilateral dual internal-external iliac occlusion produced adequate, profound, and sustained unilateral hindlimb ischemia. Because preservation of the contralateral limb as an unaffected internal control was a central design feature of the model, we elected not to occlude the contralateral IIA.

**Table I:** Comparison of approaches for inducing unilateral hindlimb ischemia in swine

Approach	Pros	Cons/limitations
Pneumatic tourniquet	Simple, rapid, noninvasive; does not require vascular access	Does not achieve sustained or complete ischemia; persistent arterial flow on angiography; residual pulse oximetry signals; inconsistent between animals
Distal aortic balloon occlusion	Produces immediate, complete ischemia of both hindlimbs; technically straightforward	Bilateral ischemia prevents use of contralateral internal control; causes systemic morbidity and postoperative paralysis; not representative of unilateral ALI
Single external iliac balloon occlusion	Minimally invasive; initially produces unilateral ischemia; technically feasible	Ischemia not durable (signals return within ∼1 hour); internal iliac collaterals reconstitute flow; insufficient to model profound ALI
Dual internal + external iliac balloon occlusion (final model)	Produces complete, sustained unilateral ischemia for 6 hours; minimally invasive; reproducible; preserves contralateral control; validated by angiography or Doppler or oximetry; minimal systemic impact	Requires two vascular access sites; balloons must remain stable throughout ischemic period

*ALI*, Acute limb ischemia.

#### Tourniquet model

A pneumatic tourniquet was applied proximal to the thigh. This produced only transient ischemia, with persistent Doppler and pulse oximetry signals in all trials, indicating incomplete arterial occlusion.

#### Distal aortic balloon occlusion

Distal aortic occlusion was performed using a 4F COBRA-OS REBOA aortic occlusion catheter (Front Line Medical Technologies, Inc), advanced into the infrarenal aorta under fluoroscopic guidance, and inflated until angiographic cessation of distal flow was achieved.

Although this resulted in complete bilateral hindlimb ischemia, animals developed significant systemic compromise, postoperative weakness, and inability to ambulate. Because the contralateral limb could not serve as an internal control and gait assessments were not feasible, this approach was abandoned.

#### Isolated external iliac artery occlusion

Isolated EIA occlusion was performed using a 4F COBRA-OS REBOA aortic occlusion catheter positioned in the ipsilateral EIA and inflated until angiographic and Doppler evidence of distal flow cessation was achieved.

**Fig 2:**
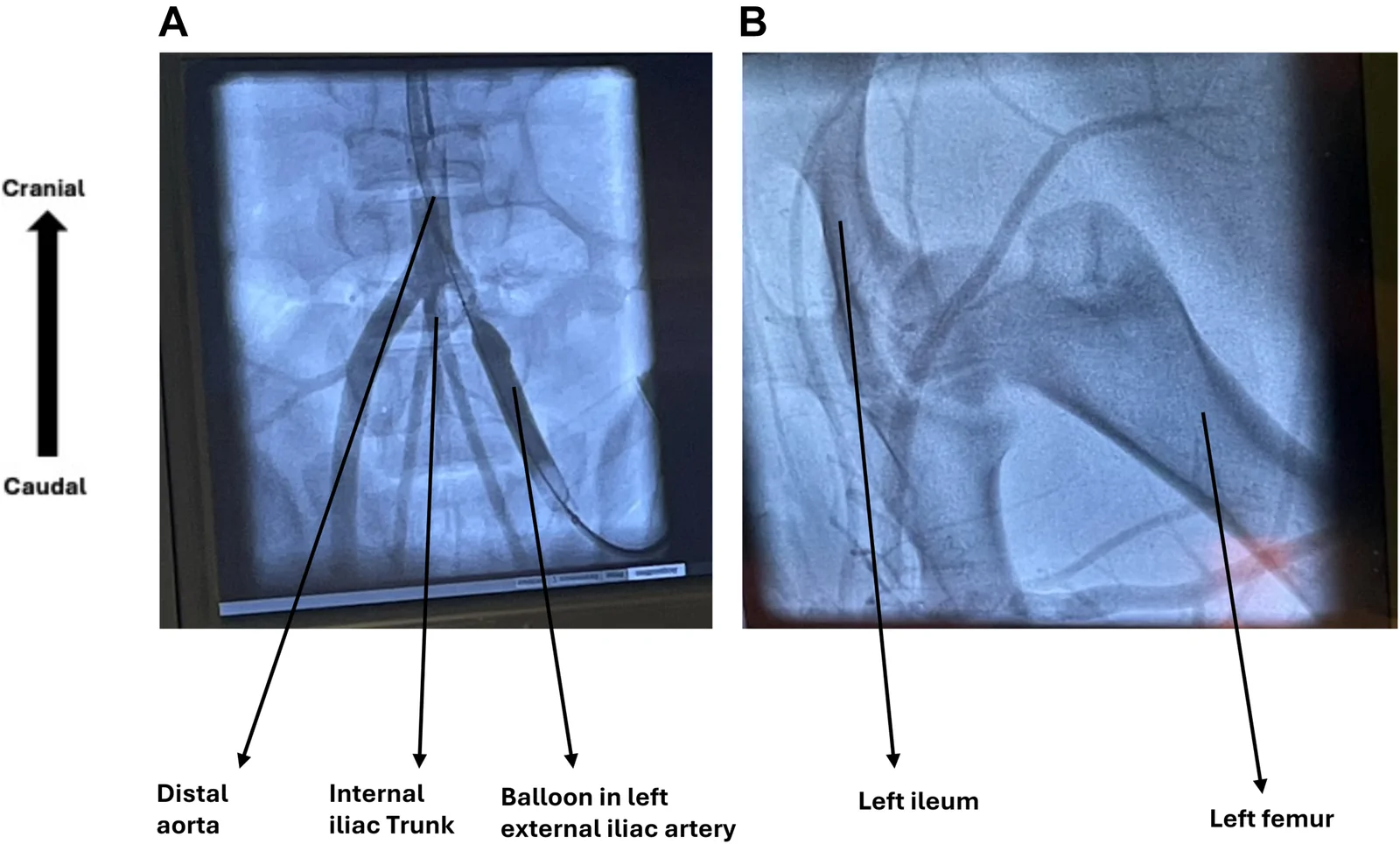
One hour after balloon occlusion of the left external iliac artery in swine, angiography demonstrated **(A)** collateral perfusion to the left hindlimb arising from the internal iliac artery trunk and **(B)** restored blood flow to the left hindlimb.

Although initial Doppler silence was achieved, distal perfusion reliably returned within ∼1 hour, as demonstrated by the reappearance of pulse oximetry signals and angiographic reconstitution of the lower-extremity arterial flow through pelvic collaterals to the groin and directly to the knee (Fig 2 , *A* and *B*). Thus, isolated EIA occlusion was insufficient for sustained ischemia.

#### Dual internal-external iliac balloon occlusion (final model)

To eliminate all major inflow sources, occlusion balloons were placed in both the ipsilateral EIA and IIA. The EIA balloon was advanced via the femoral arterial sheath, whereas the IIA balloon was introduced through the carotid arterial sheath.

The EIA was occluded using a 10 × 40 mm EverCross percutaneous transluminal angioplasty balloon catheter (Medtronic; 6F profile, 80 cm working length, 0.035″ guidewire compatible; nominal pressure 7 atm, rated burst pressure 11 atm). The IIA was occluded using a 6 × 20 mm EverCross percutaneous transluminal angioplasty balloon catheter (Medtronic; 5F profile, 80 cm working length, 0.035″ guidewire compatible; nominal pressure 8 atm, rated burst pressure 14 atm).

**Fig 3:**
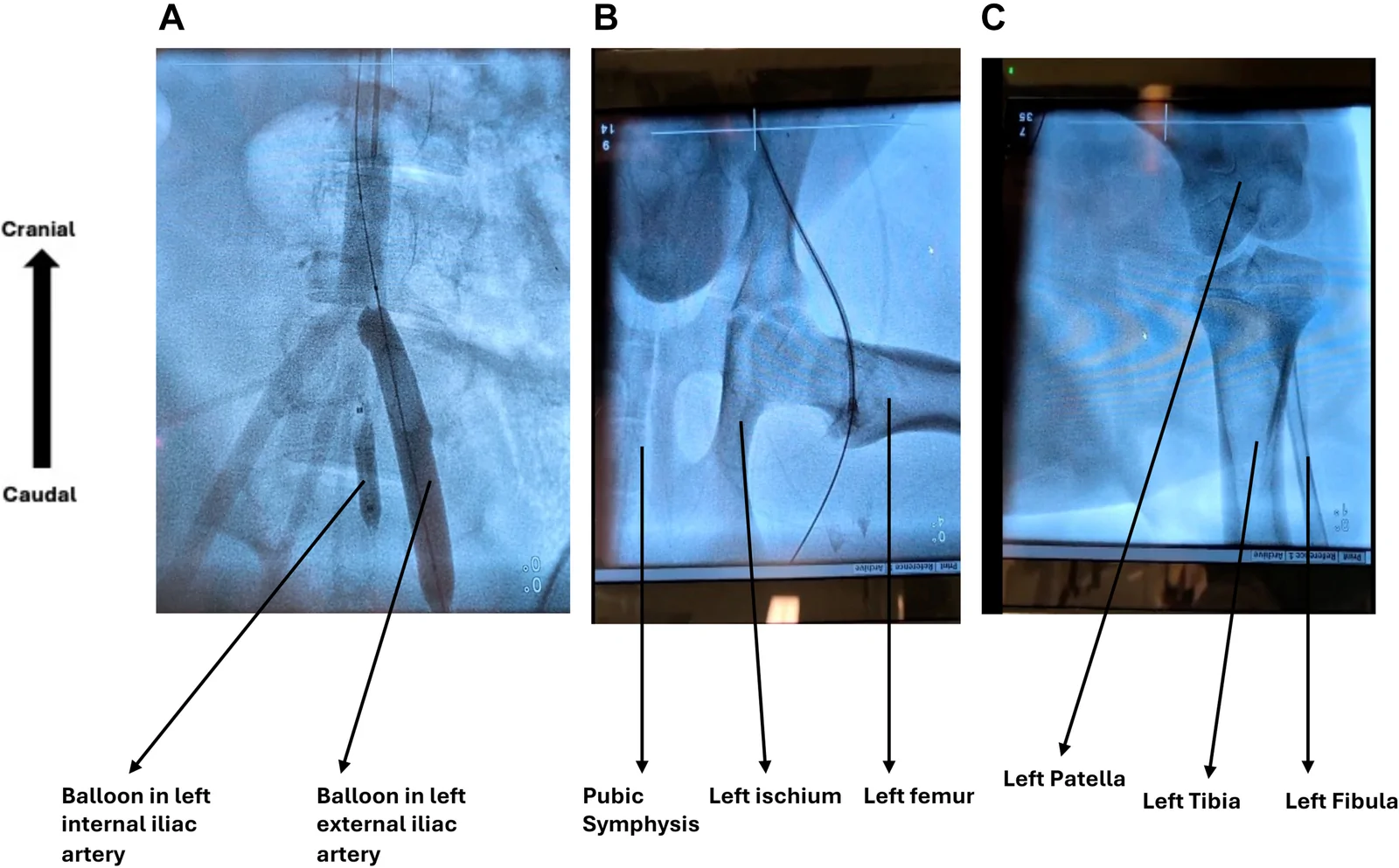
Representative angiography in swine, showing the following: **A,** Successful deployment of occlusive ballons in the left internal and external iliac arteries; **B,** lack of blood flow to the left proximal thigh; and **C,** lack of blood flow to the left proximal leg.

This configuration consistently produced complete and sustained ischemia for the full 6-hour occlusion period, with absent pulse oximetry and Doppler signals and no distal perfusion on serial angiography (Fig 3, *A-C*). This dual internal-external iliac balloon occlusion strategy was adopted as the final survival model.

### Ischemia and reperfusion protocol

After baseline angiography and functional assessments, both the EIA and IIA balloons were inflated to achieve complete occlusion. Ischemia was maintained for 6 hours, with hourly confirmation using angiography, Doppler, and pulse oximetry.

At the end of the ischemic period, calcium gluconate (100 mg/kg IV) was administered to mitigate reperfusion-associated electrolyte shifts, and the occlusion balloons were deflated to allow controlled reperfusion. Protamine sulfate (1 mg per 100 units of heparin administered) was then given intravenously for anticoagulation reversal, after which arterial sheaths were removed. Both the left carotid artery and the left internal jugular vein, each of which had been cannulated for vascular access, were ligated. The neck incision was irrigated, hemostasis confirmed, and the platysma and subcutaneous tissues were reapproximated with absorbable sutures. The skin was closed with running Monocryl. Animals recovered from anesthesia under close monitoring and were observed for 7 days, with no mortality during the ischemia, reperfusion, or survival period.

### Functional assessment

Hindlimb motor function was evaluated using the modified Tarlov score (0-4 scale), consistent with previous porcine extremity ischemia models.^9^^,^^10^ Animals were assessed at baseline, immediately postoperatively, and daily through POD 7. Longitudinal modified Tarlov scores were analyzed in animals that had complete functional assessments (baseline through POD 7). Because modified Tarlov scores are ordinal repeated-measures data, changes over time were assessed using the Friedman test. A two-sided *P* value < .05 was considered statistically significant.

### Electrophysiologic assessment

Nerve conduction studies were performed under anesthesia at baseline, immediately postoperatively, and on POD7. Compound muscle action potentials (CMAPs) were obtained by stimulation of the peroneal division of the sciatic nerve at the distal thigh and recording from the peroneus tertius muscle. Sensory nerve action potentials (SNAPs) were obtained by stimulating the medial plantar nerve in the foot and recording responses orthodromically at the ankle. Peripheral nerve stimulation was achieved with needle electrodes, and responses were recorded using transdermal electrodes to achieve precise and nonartifactual responses. Amplitude and latency values from the ischemic limb were compared with values for the contralateral internal-control limb.

### Tissue collection and histologic analysis

At POD7, animals were euthanized, and segments of the peroneus tertius muscle and tibial nerve were harvested for blinded histologic analysis. Hematoxylin and eosin (H&E)-stained sections were scored (adapted from previously published papers in this field)^9^^,^^10^ by a board-certified pathologist blinded to treatment side. Scoring scale is summarized in Table II.

**Table II:** Histopathologic scoring system for skeletal muscle and peripheral nerve injury

Parameter	0 Absent	1 Minimal (<10%)	2 Mild (10%-25%)	3 Moderate (26%-50%)	4 Marked (>50%)
Muscle inflammatory cell infiltrate	None	Rare or infrequent finding	Noticeable but not prominent finding	Prominent but not dominant finding	Dominating and/or overwhelming finding
Muscle degeneration	None	Rare or infrequent finding	Noticeable but not prominent finding	Prominent but not dominant finding	Dominating and/or overwhelming finding
Muscle necrosis	None	Rare or infrequent finding	Noticeable but not prominent finding	Prominent but not dominant finding	Dominating and/or overwhelming finding
Muscle regeneration	None	Rare or infrequent finding	Noticeable but not prominent finding	Prominent but not dominant finding	Dominating and/or overwhelming finding
Muscle edema	None	Rare or infrequent finding	Noticeable but not prominent finding	Prominent but not dominant finding	Dominating and/or overwhelming finding
Nerve degeneration	None	Rare or infrequent finding	Noticeable but not prominent finding	Prominent but not dominant finding	Dominating and/or overwhelming finding
Nerve inflammation and plasma cells	None	Rare or infrequent finding	Noticeable but not prominent finding	Prominent but not dominant finding	Dominating and/or overwhelming finding

Muscle and nerve injury parameters were graded by a blinded pathologist using hematoxylin and eosin (H&E)-stained sections and a semiquantitative 0 to 4 ordinal scale.

Muscle injury was graded according to the degree of inflammatory infiltration, myofiber degeneration, necrosis, edema, and regeneration. Nerve sections were similarly evaluated for axonal degeneration, demyelination, and inflammation. Composite injury scores were calculated for each limb.

## Results

### Distal aortic balloon occlusion causes bilateral ischemia with early severe functional deficit

Distal aortic balloon occlusion produced complete bilateral hindlimb ischemia during the occlusion period. The animal tolerated both ischemia and reperfusion without intraoperative or immediate postoperative instability and recovered uneventfully from anesthesia.

On POD1, however, the animal was alert and able to sit upright but was completely nonambulatory, with inability to bear weight or initiate stepping in either hindlimb. In accordance with predefined the Institutional Animal Care and Use Committee humane endpoints, the animal was euthanized on POD1.

Histopathologic analysis demonstrated acute bilateral ischemic muscle injury, characterized by multifocal, patchy monophasic myofiber necrosis with associated hemorrhage and neutrophilic infiltration, consistent with acute ischemia-reperfusion injury.

Because distal aortic occlusion resulted in bilateral limb dysfunction, precluded use of a contralateral internal control, and prevented longitudinal functional assessment, this approach was abandoned.

### External iliac-only occlusion fails to produce sustained ischemia

Isolated balloon occlusion of the EIA produced only transient ischemia. Distal perfusion initially ceased, but pulse oximetry and Doppler signals returned within ∼1 hour. Repeat angiography confirmed persistent EIA balloon occlusion with absent antegrade flow, yet demonstrated rapid collateral reconstitution of the femoral circulation via extensive internal-iliac pathways (Fig 2, *A* and *B*). Functionally, animals ambulated normally on POD1 (modified Tarlov score, 4). Histology showed rare, minimal myofiber necrosis (<10%), with all other muscle and nerve parameters within normal limits.

### Dual internal-external iliac occlusion produces complete, durable ischemia

Simultaneous occlusion of the ipsilateral internal and EIAs reliably produced complete, sustained hindlimb ischemia. Pulse oximetry and Doppler remained absent throughout the 6-hour occlusion, and serial angiography confirmed no distal perfusion (Fig 3, *B* and *C*). All animals survived to endpoint without major systemic complications.

### Functional, electrophysiologic, and tissue-level outcomes

**Fig 4:**
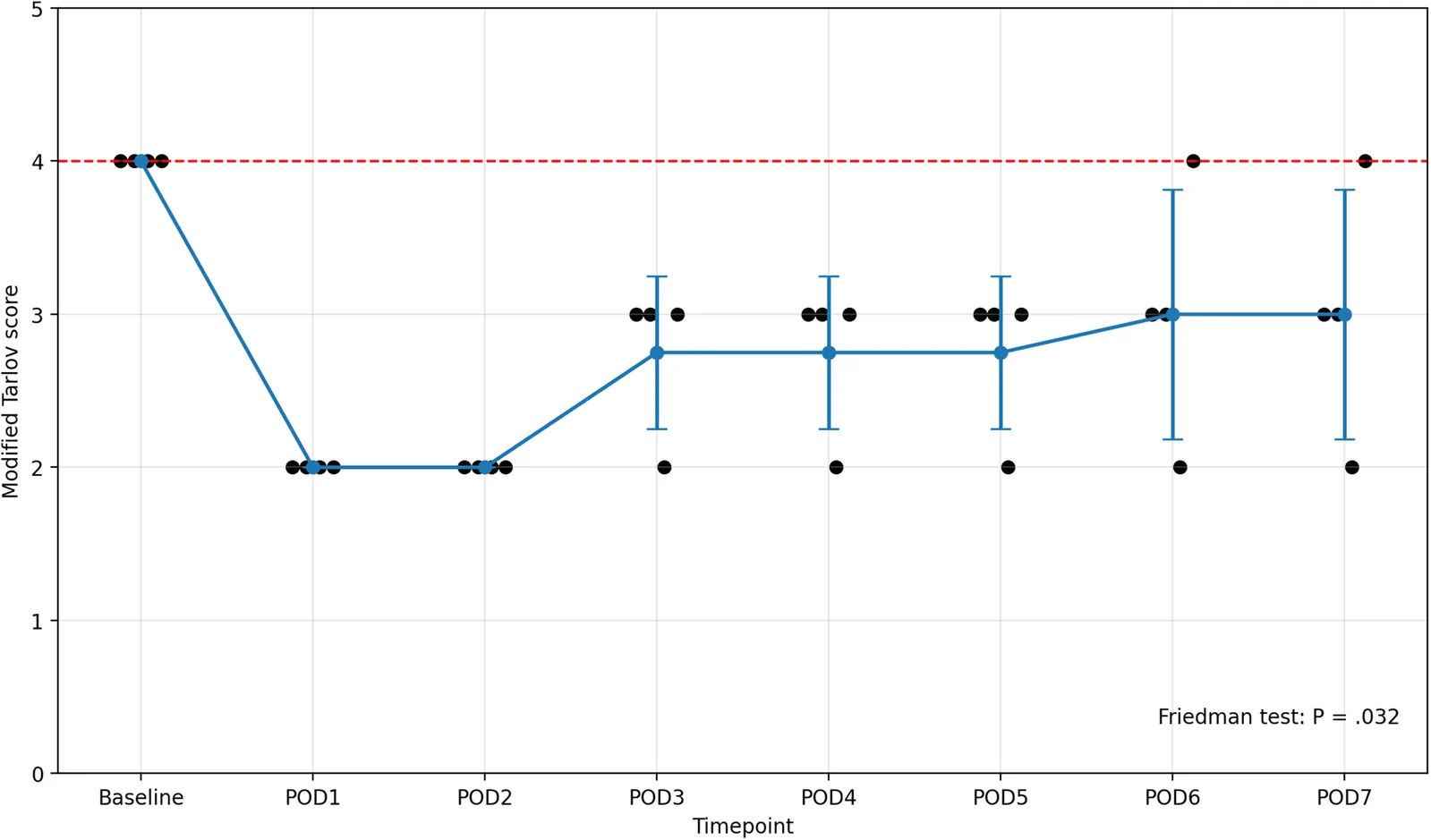
Modified Tarlov gait scores across baseline and postoperative days (*POD*) 1 to 7 in swine that underwent dual internal-external iliac balloon occlusion with complete longitudinal functional assessment (n = 4). Scores reflect global ambulatory function after unilateral hindlimb ischemia. Data are shown as means ± SD and overlaid points represent individual animals. The *red dashed line* indicates baseline normal gait score. Longitudinal change over time was assessed using the Friedman test (*χ*^2^ = 15.31; df = 7; *P* = .032).

Modified Tarlov scores declined from 4.0 at baseline to 2.0 by POD1-2, indicating standing without weight bearing. Scores partially recovered from POD3-7 but remained below baseline in most animals at 1 week. Longitudinal change in gait score over time was statistically significant by the Friedman test (*χ*^2^ = 15.31; *d*_*f*_ = 7; *P* = .032; Fig 4).

**Fig 5:**
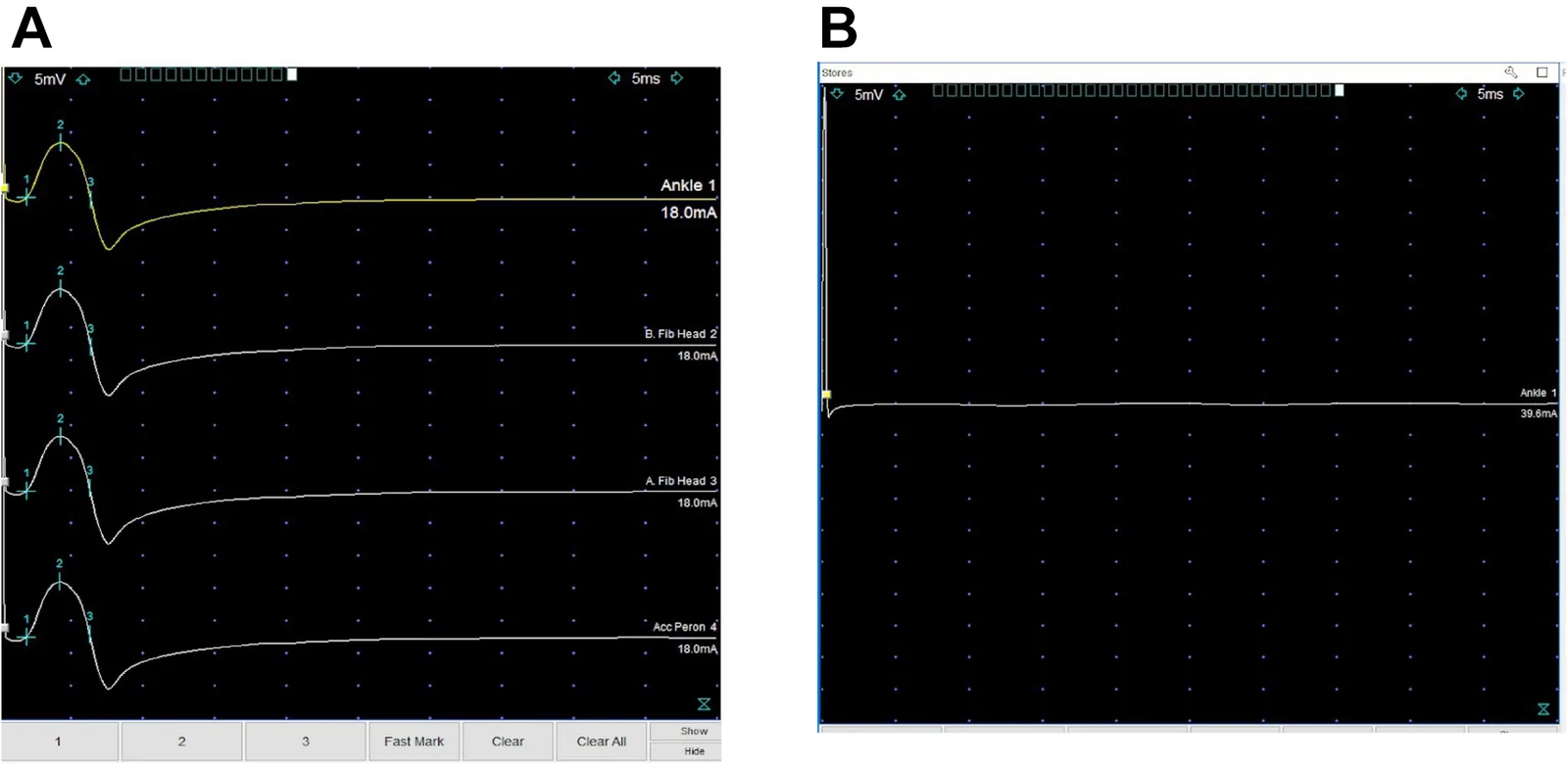
Representative motor nerve conduction tracing in swine from the ischemic limb at baseline and postoperative day 7 (POD7). **A,** Baseline tracing showing reproducible compound muscle action potentials (CMAPs) after stimulation of the peroneal division of the sciatic nerve. **B,** POD7 tracing showing absent CMAP responses despite stimulation, consistent with severe neuromuscular dysfunction following ischemia-reperfusion injury.

**Fig 6:**
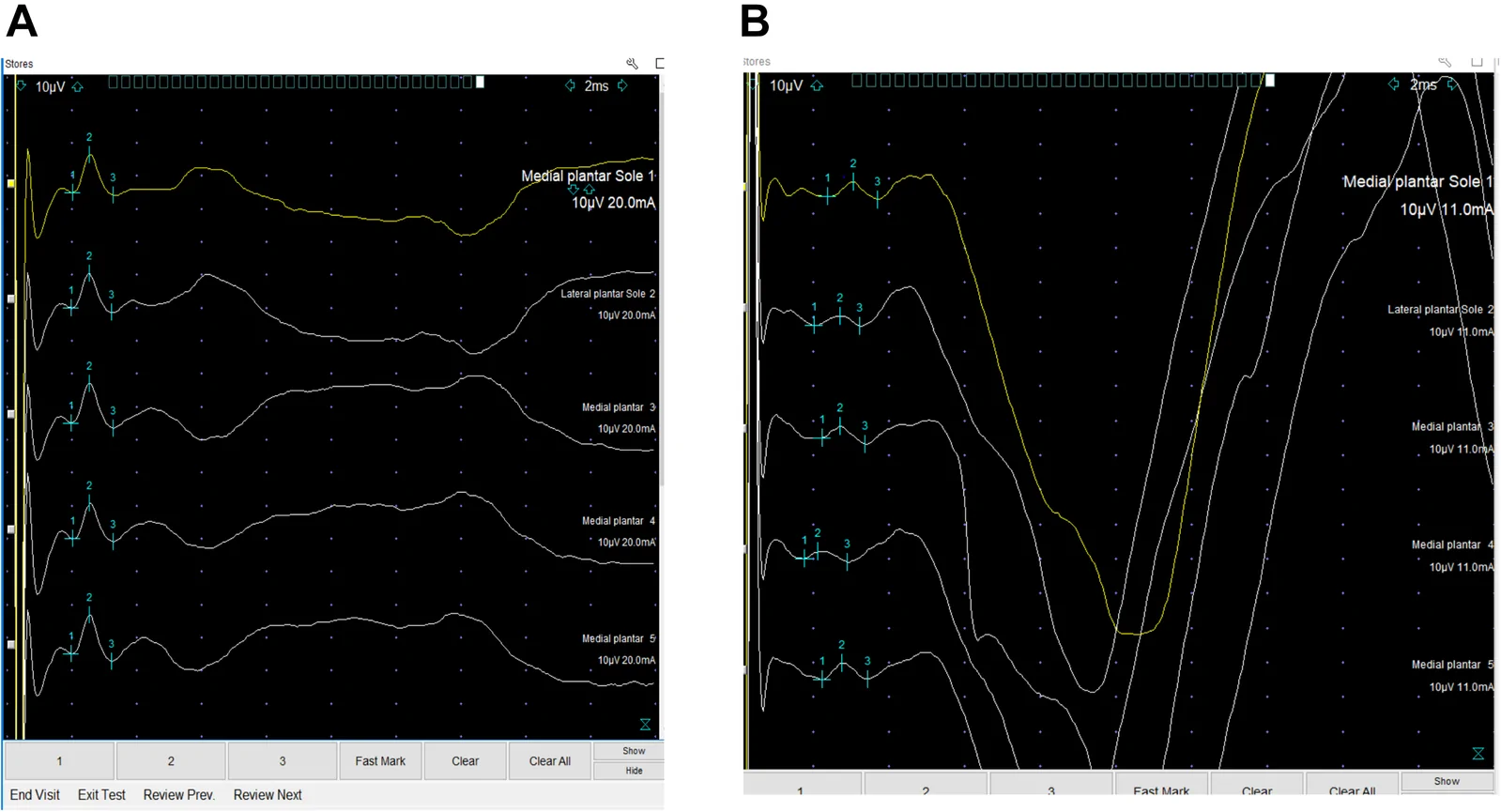
Representative sensory nerve conduction tracing in swine from the ischemic limb at baseline and postoperative day 7 (POD7). **A,** Baseline tracing showing reproducible sensory nerve action potentials (SNAPs) after stimulation of the medial plantar nerve. **B,** POD7 tracing showing near-absent SNAP responses despite stimulation, consistent with severe peripheral nerve dysfunction following ischemia-reperfusion injury.

**Fig 7:**
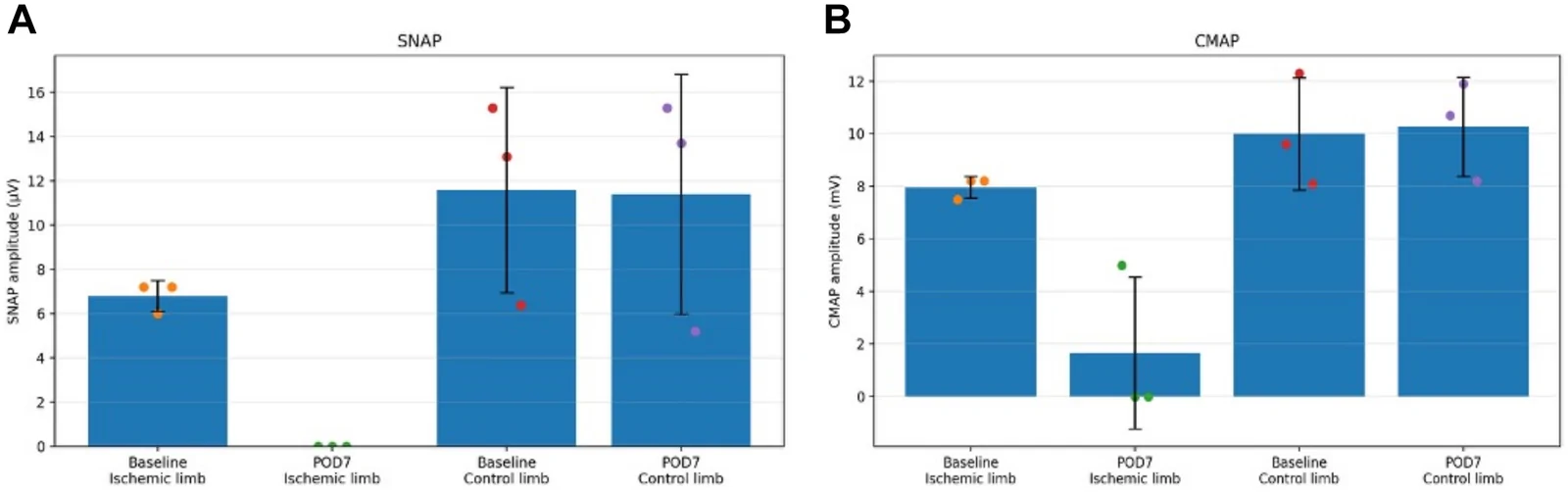
Nerve conduction studies following unilateral hindlimb ischemia in swine. **A,** Sensory nerve action potential (*SNAP*) amplitudes (μV). **B,** Compound muscle action potential (*CMAP*) amplitudes (mV). SNAP and CMAP amplitudes were measured in the ischemic and contralateral control limbs at baseline and postoperative day 7 (*POD7*) in animals with complete electrophysiologic testing (n = 3). *Bars* represent means ± SD, and overlaid points represent individual animals. At baseline, SNAP and CMAP amplitudes were preserved in both limbs. By POD7, SNAP amplitude was absent in the ischemic limb in all animals, whereas responses remained preserved in the contralateral control limb. CMAP amplitudes similarly declined markedly in the ischemic limb at POD7, consistent with severe neuromuscular injury, whereas the control limb maintained preserved motor responses. Given the limited sample size, these outcomes were summarized descriptively, and formal inferential statistical testing was not performed. *SD*, Standard deviation.

Figs 5 and 6 provide representative nerve conduction tracings demonstrating near-complete loss of compound muscle action potentials (CMAPs) and SNAPs in the ischemic limb from baseline to POD7. Electrophysiologic testing was completed in three animals with paired baseline and POD7 assessments of the ischemic and contralateral control limbs. Quantitative group-level amplitudes are summarized descriptively in Fig 7. At baseline, SNAP and CMAP amplitudes were preserved in both limbs. By POD7, SNAP amplitude was absent in the ischemic limb in all animals, whereas responses remained preserved in the contralateral control limb. CMAP amplitudes also declined markedly in the ischemic limb by POD7, whereas the contralateral control limb maintained preserved motor responses. Given the limited sample size, these electrophysiologic outcomes were summarized descriptively without formal inferential statistical testing.

**Fig 8:**
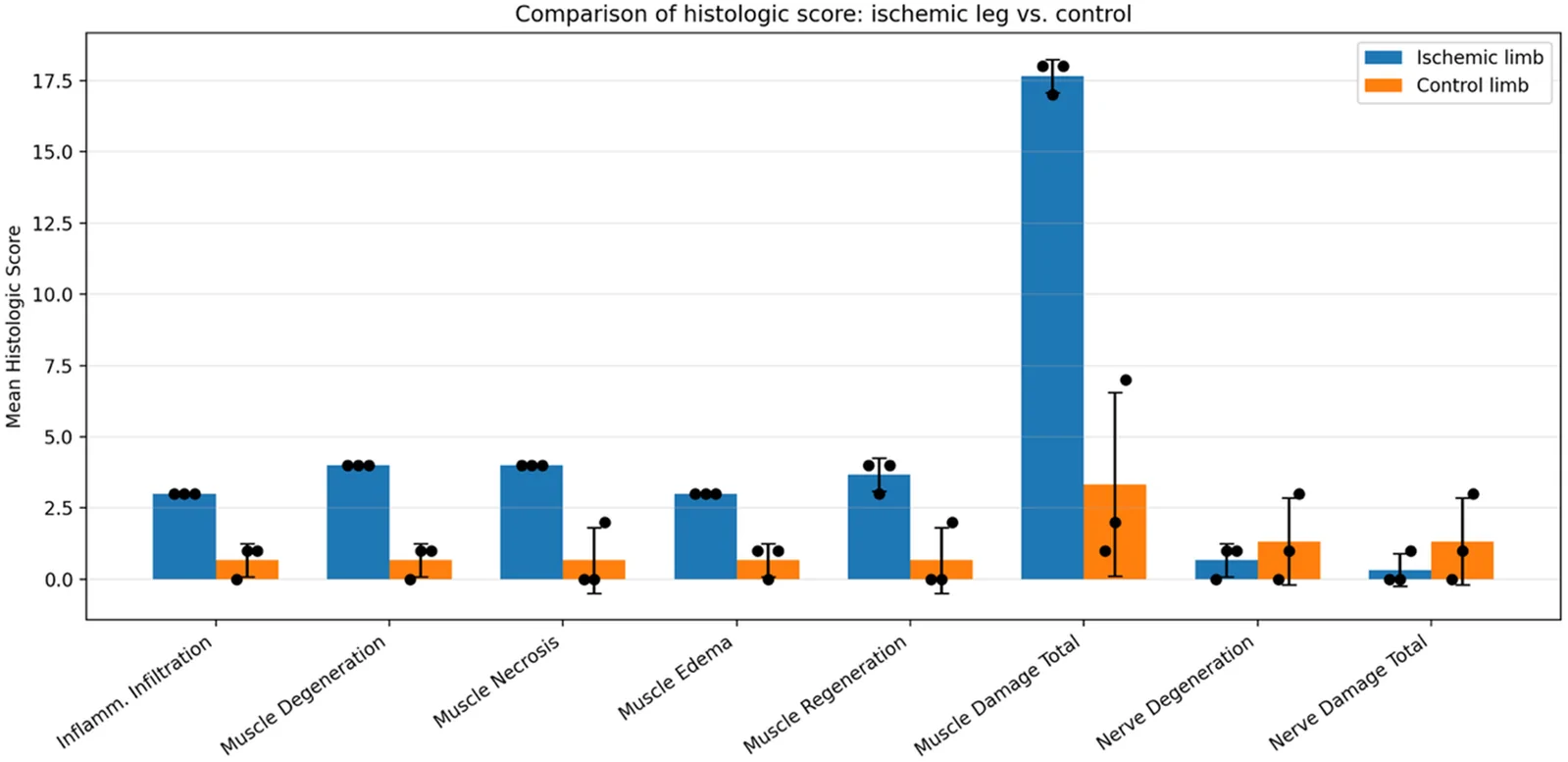
Histologic injury scores in ischemic and contralateral control limbs in swine. Means ± SD histologic scores are shown for individual muscle and nerve injury parameters in ischemic and contralateral control limbs at postoperative day 7 and overlaid points represent individual animals. Scores were higher in ischemic limbs than contralateral controls across all muscle injury domains, including inflammatory cell infiltration, muscle degeneration, muscle necrosis, edema, regeneration, and cumulative muscle damage. Nerve injury scores remained low in both groups, indicating relative neural preservation. Given the limited number of paired histologic specimens, these outcomes were summarized descriptively and formal inferential statistical testing was not performed. *SD*, Standard deviation.

**Fig 9:**
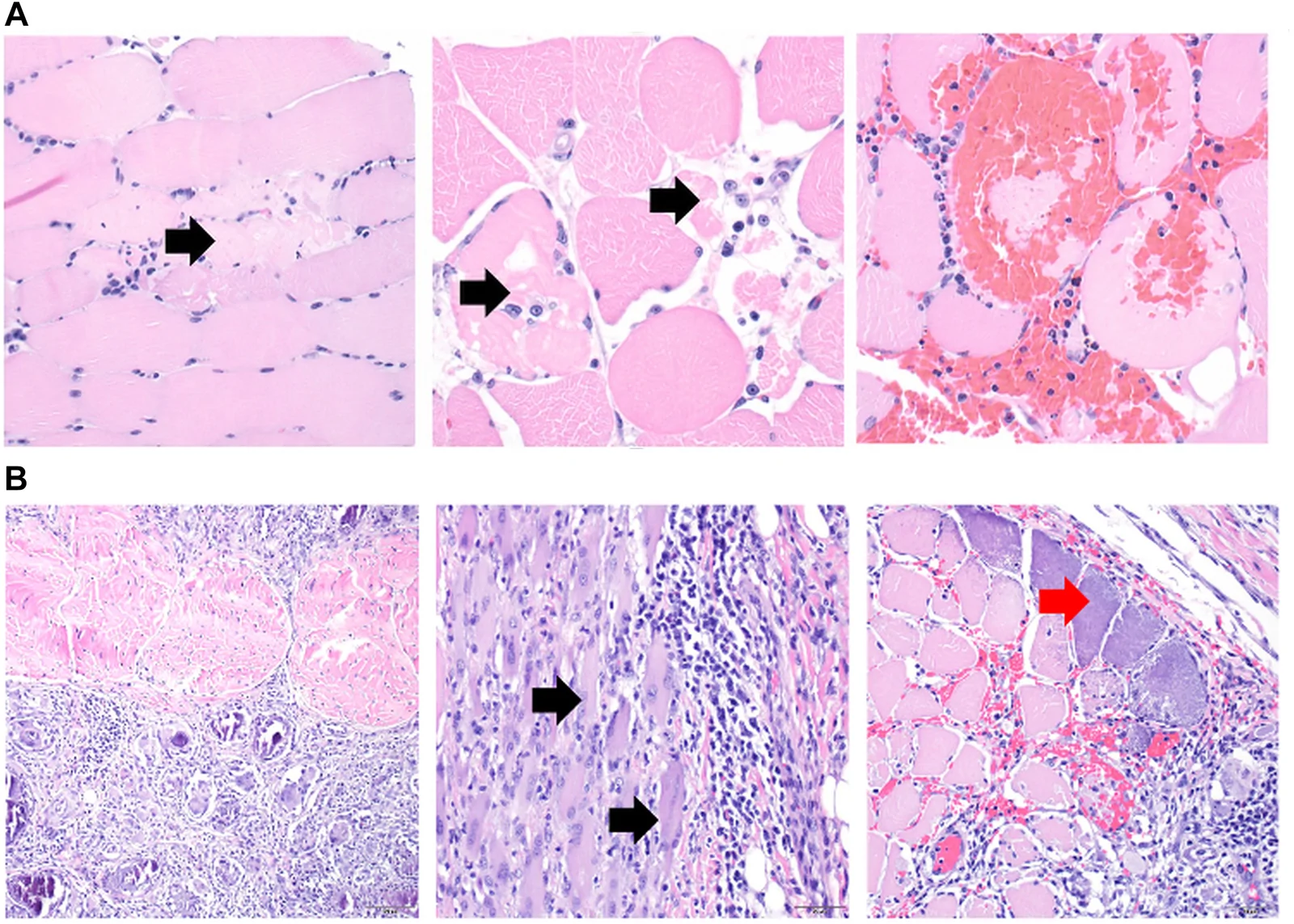
Representative hematoxylin and eosin (H&E) histology of contralateral control and ischemic skeletal muscle at postoperative day 7. **A,** Contralateral control limb demonstrating preserved myofiber architecture with minimal inflammatory infiltration. **B,** Ischemic limb demonstrating extensive injury, including diffuse myofiber degeneration, inflammatory infiltration, hemorrhage, and widespread coagulative necrosis with myofiber mineralization. *Black arrows* indicate inflammatory infiltration and myofiber injury. *Red arrow* indicates myofiber mineralization.

Quantitative histology demonstrated greater muscle injury in ischemic limbs compared with contralateral control limbs across all muscle injury domains, including inflammatory cell infiltration, muscle degeneration, muscle necrosis, edema, and regeneration. The cumulative muscle damage score was markedly higher in ischemic limbs than in contralateral controls. In contrast, nerve degeneration, nerve inflammation, and cumulative nerve damage scores remained low in both limbs, indicating relative neural preservation at POD7. Given the limited number of paired histologic specimens, these outcomes were summarized descriptively without formal inferential statistical testing (Fig 8). Representative H&E images supported the semiquantitative histologic findings. Contralateral control skeletal muscle demonstrated preserved myofiber architecture with minimal inflammatory infiltration, whereas ischemic limbs demonstrated extensive injury, including diffuse myofiber degeneration, inflammatory infiltration, hemorrhage, and coagulative necrosis with myofiber mineralization (Fig 9).

### Interpretation of the nerve conduction studies-histology mismatch

Although compound motor and SNAPs were absent, nerve H&E at POD7 was largely unremarkable. Early ischemic injury can cause axonal swelling and paranoidal dysfunction and resulting failure of electrical conduction (neuropraxia) that is indistinguishable from axonotmesis. Because routine H&E is insensitive to early demyelination or paranodal injury, and Wallerian degeneration often evolves beyond 1 week, minimal structural nerve findings at POD7 are expected. Future work will extend survival to ≥14 days and incorporate electron microscopy to resolve the temporal relationship between functional deficits and structural nerve injury. Muscle, conversely, develops myofiber necrosis within 2 to 4 hours and this muscle injury will independently contribute to the failure of compound muscle action potential generation.

### Summary

Collectively, these results demonstrate that dual internal-external iliac balloon occlusion yields a reproducible, profound ischemic insult with concordant functional, electrophysiologic, and histopathologic features of ALI and reperfusion injury.

## Discussion

In this study, we developed and validated a novel, minimally invasive porcine survival model of acute hindlimb ischemia using simultaneous endovascular balloon occlusion of the EIA and IIA. Our model overcomes two major limitations of existing large-animal ALI models. First, models relying on tourniquets or aortic cross-clamping are poorly suited for survival studies: aortic occlusion produces severe systemic and pelvic ischemia with nonambulatory postoperative animals, whereas tourniquets, in our hands, failed to generate reliable and sustained limb ischemia despite multiple configurations. Second, survival models based on single-segment ligation of the CFA or EIA are primarily designed to mimic chronic limb-threatening ischemia and are inadequate for producing profound acute ischemia because robust pelvic collaterals rapidly reconstitute distal flow.

A detailed comparison of our model with existing porcine ischemia models helps clarify the specific gaps addressed by the dual internal-external iliac balloon occlusion approach.

1 Single-segment ligation yields inadequate acute ischemia due to collateral flow: Survival models based on CFA or EIA ligation create an initial ischemic insult but fail to produce sustained, profound ischemia because internal-iliac pelvic collaterals remain patent. El Masry et al^11^ ligated and excised approximately 1″ of the CFA and confirmed diminished perfusion on day 0 using a single snapshot angiographic assessment, consistent with their goal of establishing a chronic ischemia model. However, a momentary absence of antegrade flow does not ensure durable or complete ischemia in swine.

Importantly, our serial angiography, supported by Doppler and pulse oximetry, demonstrated that when only the EIA or CFA is occluded, distal perfusion reliably returns within approximately 1 hour through robust pelvic collateral pathways. A potential counterargument is that excising the CFA segment, as done by El Masry et al,^11^ should eliminate internal-iliac inflow because many collaterals classically converge at the groin. However, our angiographic studies revealed that swine possess additional collateral channels that bypass the groin entirely, arising from branches proximal to the iliac bifurcation and re-entering the limb distally at the thigh or even near the knee. These include profunda-pelvic anastomoses, lumbar branches, and distal muscular collaterals that remain fully patent even when the CFA is ligated or excised. Consequently, CFA or EIA ligation alone does not eliminate all major inflow sources, and distal reperfusion occurs reliably unless both the IIA and EIA are simultaneously occluded.

This observation is consistent with previous mapping studies. Gao et al^12^ demonstrated variable but substantial collateral development through the internal-iliac network and its proximal branches following arterial ligation. The underlying anatomy explains this phenomenon: pigs lack common iliac arteries; instead, the distal aorta trifurcates into both external iliacs and a single, dominant internal-iliac trunk that gives rise to extensive proximal pelvic and lumbar branches capable of reconstituting distal perfusion. Because single-segment ligation leaves these pathways intact, it cannot generate the depth or duration of ischemia required to model true ALI.

2 Endovascular occlusion without internal-iliac control leaves residual inflow: Endovascular approaches, including embolization or balloon occlusion of a single iliac artery, reliably induce acute ischemia but suffer from the same anatomic limitation as single-segment ligation: persistent internal-iliac collateral inflow that reconstitutes distal perfusion. Kim et al^13^ used intravascular embolization of the EIA and noted partial inflow persistence when internal-iliac supply remained patent. Consequently, ischemia depth is limited, and the model cannot replicate the severity or reproducibility required for mechanistic ALI studies.

A potential alternative endovascular configuration would be ipsilateral EIA occlusion combined with bilateral IIA occlusion to further restrict pelvic collateral inflow. We considered this approach; however, our angiographic observations demonstrated that leaving the contralateral IIA patent did not result in appreciable collateral reconstitution to the ischemic limb when the ipsilateral IIA and EIAs were simultaneously occluded. In contrast, bilateral internal iliac occlusion would introduce unnecessary risk of pelvic ischemia and related complications, including bowel, bladder, and gluteal ischemia, which could confound postoperative recovery and undermine the goals of a survival model.

Accordingly, we selected ipsilateral dual internal-external iliac balloon occlusion as the most balanced strategy, achieving complete and sustained limb ischemia while minimizing systemic and pelvic morbidity.

3 Abdominal aortic/junctional tourniquet models are nonsurvival and systemically confounded: Tourniquet-based and abdominal aortic/junctional tourniquet models reliably induce rapid, global ischemia and are useful for short-term physiologic or hemorrhage-control studies. However, they are inherently nonsurvival models because they produce substantial systemic metabolic stress, rhabdomyolysis, and cardiovascular instability. Bilateral ischemia also precludes use of the contralateral limb as an internal control and prevents meaningful assessment of gait or delayed neuromuscular recovery. These limitations make them unsuitable for controlled ischemia-reperfusion studies requiring survival and longitudinal follow-up.
4 Advantages of the dual internal-external iliac balloon occlusion model: Our dual-occlusion strategy eliminates these limitations by simultaneously occluding both the EIA and IIA, thereby removing all major inflow pathways to the limb. This produces uniform, complete, and sustained unilateral ischemia for 6 hours without systemic compromise. Because the contralateral limb remains unaffected, functional, electrophysiologic, and histopathologic comparisons can be made within the same animal—substantially increasing model power, reliability, and translational utility.

The primary limitations of this model relate to its ability to fully recapitulate the heterogeneity and clinical context of human acute lower limb ischemia, which may arise from embolic occlusion, in situ thrombosis, peripheral arterial disease, trauma, or vascular intervention, often in patients with atherosclerosis, endothelial dysfunction, and variable collateralization. In contrast, this model used otherwise healthy swine and produced a controlled, abrupt, unilateral inflow occlusion by simultaneous endovascular balloon occlusion of the IIA and EIA. Porcine hindlimb collateral circulation also differs from human collateral anatomy and may influence the degree and distribution of ischemic injury. In addition, porcine skeletal muscle and peripheral nerve may be relatively more tolerant of ischemic insult than human tissue, which may affect the timing and severity of injury observed in this model. These factors limit direct extrapolation to all forms of human ALI. However, the ability of this model to produce reproducible functional, histologic, and neuromuscular injury despite these collateral and species-related differences supports its utility as a clinically relevant platform for studying ischemia-reperfusion injury and evaluating limb-protective interventions.

The 6-hour occlusion interval was selected to produce a severe, reproducible ischemic insult in swine that would result in measurable functional, electrophysiologic, and histopathologic injury at 7 days while still permitting survival assessment. This duration should not be interpreted as directly equivalent to 6 hours of untreated ischemia in patients, because porcine skeletal muscle may tolerate warm ischemia differently than human skeletal muscle. Although a shorter occlusion period may produce milder injury, it may not reliably generate the durable deficits needed for this survival model. Accordingly, this model is best viewed as a reproducible platform for studying controlled unilateral ischemia-reperfusion injury and candidate therapies rather than a complete simulation of all forms of human acute lower limb ischemia.

Histopathologic assessment at POD 7 captures early ischemic injury but may not fully reflect delayed nerve degeneration, fibrosis, or long-term functional remodeling seen in clinical ALI. Histologic characterization in this initial model development study was limited to blinded H&E assessment of skeletal muscle and peripheral nerve. Immunohistochemistry was not performed because the primary objective was to establish the feasibility and reproducibility of the survival model using standard morphologic injury endpoints, rather than to define specific molecular pathways of ischemia-reperfusion injury. We recognize that immunohistochemical markers of inflammation, myocyte injury and regeneration, microvascular injury, axonal injury, and demyelination could provide additional mechanistic insight. Future studies using this model will extend survival duration and incorporate specialized immunohistochemistry and ultrastructural analysis to better characterize the temporal evolution and mechanisms of muscle and nerve injury after reperfusion.

Despite these limitations, the model provides a reproducible survival platform for studying controlled unilateral ischemia-reperfusion injury with longitudinal functional, electrophysiologic, and histopathologic assessment. One motivation for developing this model was to establish a large-animal platform for testing candidate limb-salvage therapies after ALI. Because it produces durable unilateral injury while preserving the contralateral limb as an internal control, this model may be useful for evaluating reperfusion strategies, pharmacologic therapies, device-based interventions, and targeted protective approaches such as therapeutic limb hypothermia.

## Conclusions

In this study, we systematically evaluated multiple large-animal strategies for inducing acute hindlimb ischemia, including tourniquet-based, aortic, single-segment iliac, and endovascular occlusion approaches, and identified key limitations that restrict their utility for survival and longitudinal assessment. Through this iterative process, we demonstrate that simultaneous endovascular occlusion of the IIA and EIA provides a reproducible, survival porcine model of profound unilateral ischemia that overcomes collateral-driven limitations of previous approaches. This model enables controlled ischemia-reperfusion injury with longitudinal functional, electrophysiologic, and histopathologic evaluation, supporting its use in translational studies of ALI.

## Author Contributions

Conception and design: AT, SP, TM, PD, ZZ, DB, PR, RR, SA

Analysis and interpretation: AT, SP, SA, TM, PD, PR

Data collection: AT, SP, TM, PD, ZZ, DB, SA

Writing the article: AT, SP, TM, ZZ, DB, RR, SA

Critical revision of the article: AT, SA, TM, PD, PR

Final approval of the article: AT, SP, TM, PD, ZZ, DB, PR, RR, SA

Statistical analysis: AT, SA

Obtained funding: SA

Overall responsibility: SA

## Funding

This work was supported by research and development funding from the 10.13039/100000005U.S. Department of Defense, including 10.13039/100006831Air Force 10.13039/100015412Small Business Technology Transfer (STTR) Phase I and Phase II awards and an Army 10.13039/100000090Congressionally Directed Medical Research Programs (10.13039/100000090CDMRP) contract. Additional early-stage development support was provided through institutional grant funding.

## Disclosures

None.

## References

[1] Rosenberg H., Rosenberg E., Kubelik D. (2023). Acute limb ischemia. CMAJ.

[2] Jarosinski M.C., Kennedy J.N., Iyer S. (2025). Contemporary National incidence and outcomes of acute limb ischemia. Ann Vasc Surg.

[3] Mustapha J.A., Katzen B.T., Neville R.F. (2018). Determinants of long-term outcomes and costs in the management of critical limb ischemia: a population-based cohort study. J Am Heart Assoc.

[4] Björck M., Earnshaw J.J., Acosta S. (2020). Editor’s choice—European Society for Vascular Surgery (ESVS) 2020 clinical practice guidelines on the management of acute limb ischaemia. Eur J Vasc Endovasc Surg.

[5] Luengo-Fernandez R., Howard D.P.J., Nichol K.G., Dobell E., Rothwell P.M. (2018). Hospital and institutionalisation care costs after limb and visceral ischaemia benchmarked against stroke: long-term results of a population based cohort study. Eur J Vasc Endovasc Surg.

[6] Morris S.F., Pang C.Y., Zhong A., Boyd B., Forrest C.R. (1993). Assessment of ischemia-induced reperfusion injury in the pig latissimus dorsi myocutaneous flap model. Plast Reconstr Surg.

[7] Picard-Ami L.A., Thomson J.G., Kerrigan C.L. (1990). Critical ischemia times and survival patterns of experimental pig flaps. Plast Reconstr Surg.

[8] Eckert P., Schnackerz K. (1991). Ischemic tolerance of human skeletal muscle. Ann Plast Surg.

[9] Hancock H.M., Stannard A., Burkhardt G.E. (2011). Hemorrhagic shock worsens neuromuscular recovery in a porcine model of hind limb vascular injury and ischemia-reperfusion. J Vasc Surg.

[10] Burkhardt G.E., Gifford S.M., Propper B. (2011). The impact of ischemic intervals on neuromuscular recovery in a porcine (Sus scrofa) survival model of extremity vascular injury. J Vasc Surg.

[11] El Masry M.S., Gnyawali S.C., Sen C.K. (2023). Robust critical limb ischemia porcine model involving skeletal muscle necrosis. Sci Rep.

[12] Gao Y., Aravind S., Patel N.S. (2020). Collateral development and arteriogenesis in hindlimbs of swine after ligation of arterial inflow. J Surg Res.

[13] Kim W., Choi D., Jang Y., Nam C.M., Hur S.H., Hong M.K. (2020). Effect of intentional restriction of venous return on tissue oxygenation in a porcine model of acute limb ischemia. PLoS One.

